# First person – Roza Masalmeh

**DOI:** 10.1242/dmm.052677

**Published:** 2025-11-28

## Abstract

First Person is a series of interviews with the first authors of a selection of papers published in Disease Models & Mechanisms, helping researchers promote themselves alongside their papers. Roza Masalmeh is first author on ‘ FAK modulates glioblastoma stem cell energetics via regulation of glycolysis and glutamine oxidation’, published in DMM. Roza is a postdoc in the lab of Margaret Frame at Institute of Genetics and Cancer, The University of Edinburgh, Western General Hospital, Edinburgh, UK, investigating brain tumour vulnerabilities, particularly metabolic and cell–adhesion dependencies, to guide better treatments.


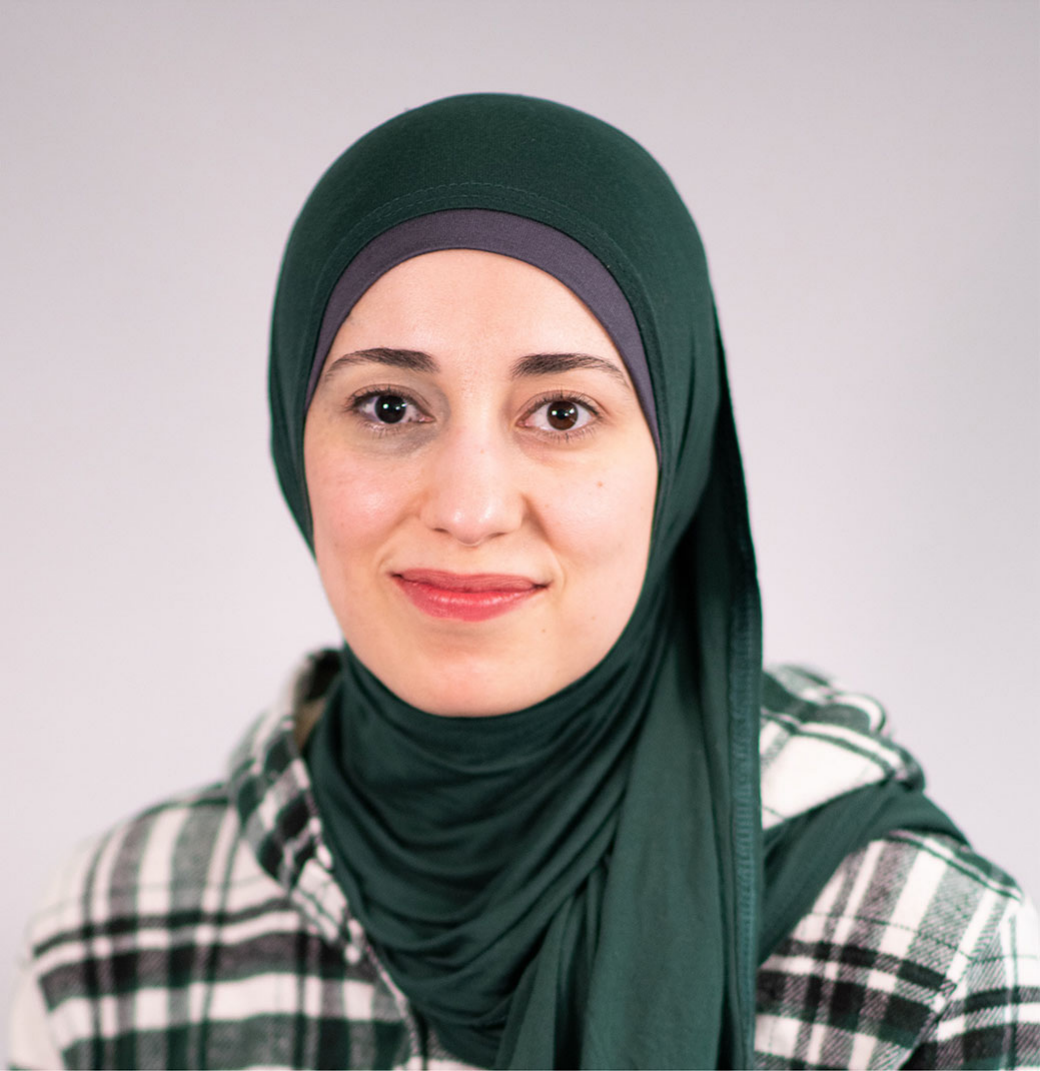


**Roza Masalmeh**

**Who or what inspired you to become a scientist?**

I've been drawn to science from an early age. I did small experiments at home – once even trying to fuse two metals when my parents weren't looking – and spent hours observing ants to understand how they communicated and behaved. At school, I loved lessons that centred on experiments and observations, and I joined the science club, where we carried out small projects, like building model hot-air balloons and studying anatomy. As I grew up, I knew that, on the one hand, I liked science and, on the other hand, I liked helping people. When my aunt died of cancer, it sharpened my focus; since then, I've known this is the disease I want to research.

**What is the main question or challenge in disease biology you are addressing in this paper? How did you go about investigating your question or challenge?**

Cancer cells need a family of proteins called adhesion proteins to move and invade. The question our paper addressed was whether we could exploit glioblastoma adhesion dependencies to better treat it. Genetically and pharmacologically, I targeted a protein called focal adhesion kinase (FAK, also known as PTK2) in a glioblastoma stem–cell model. This intervention reduced cellular invasiveness *in vitro* and *in vivo*, and improved survival in a murine model. Alongside these changes, I observed a colour change in the medium in which the cells were growing, prompting the question: “Does this protein modulate metabolism?” I thought this was interesting because FAK isn't a metabolic protein. I used various techniques, including Seahorse assays, metabolomics, proteomics and super–resolution imaging, to investigate this question and uncover a mechanistic link. I found that focal adhesion kinase enhances the two main energy–producing pathways, i.e. glycolysis and oxidative phosphorylation, via mechanotransduction.

**How would you explain the main findings of your paper to non-scientific family and friends?**

Cancer cells use a family of proteins to crawl through the body. I studied one of these proteins, i.e. focal adhesion kinase (FAK), and found that, in addition to helping cells move, FAK also boosts their energy. When this protein is switched on, it helps cells grip and pull on their surroundings, making them spread and enabling them to make more energy. When we switch this protein off, the cells loosen their grip on their surroundings and, instead, pull hard on one another – they become rounder, move less and produce less energy. When we switch FAK off in mice with glioblastoma (a fast-growing brain cancer), their tumours are smaller, the cancer cells move less inside the brain and the mice live longer.

**What are the potential implications of these results for disease biology and the possible impact on patients?**

Our work suggests that combining FAK targeting with agents that inhibit residual metabolic pathways in glioblastoma may lead to greater therapeutic benefit for patients.

**Figure:**
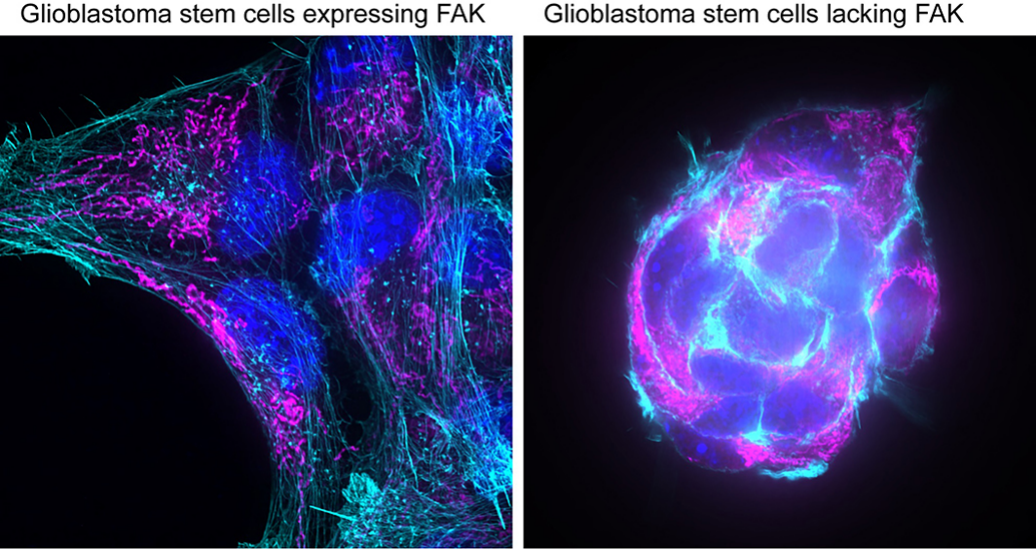
**Images show transformed stem-cell models of glioblastoma cells that either express (left) or lack (right) focal adhesion kinase (FAK).** The F-actin cytoskeleton was immunolabelled with fluorophore-conjugated phalloidin (cyan), mitochondria were stained with MitoTracker Deep Red FM (magenta), nuclei were stained with DAPI (blue).

**Why did you choose DMM for your paper?**

DMM is an excellent, fully open-access journal that publishes rigorous science; I enjoy reading its articles. It's highly relevant to my work, with a strong emphasis on translational relevance. Moreover, DMM is a partner of Review Commons, which allowed me to transfer referee reports and responses, making the publishing process more efficient.

**Given your current role, what challenges do you face and what changes could improve the professional lives of other scientists in this role?**

One challenge is staying focused on doing good science while also applying for funding to become independent. Early-career bridge funding would give me – and postdocs at a similar stage in our careers – more time to produce more high-quality papers to support our grant applications.

**What's next for you?**

Starting my independent research group focused on leveraging what we know about adhesion proteins and metabolic vulnerabilities in GBM to find better cures.

**Tell us something interesting about yourself that wouldn't be on your CV**

I enjoy chess, hiking and reading – ‘The Four Winds’ by Kristin Hannah is a favourite.
